# Why Do Data Users Say Health Care Data Are Difficult to Use? A Cross-Sectional Survey Study

**DOI:** 10.2196/14126

**Published:** 2019-08-06

**Authors:** Ho Heon Kim, Bora Kim, Segyeong Joo, Soo-Yong Shin, Hyo Soung Cha, Yu Rang Park

**Affiliations:** 1 Department of Biomedical Systems Informatics Yonsei University College of Medicine Seoul Republic of Korea; 2 Emphasis Information Technology Inc Seoul Republic of Korea; 3 Department of Biomedical Engineering University of Ulsan College of Medicine Seoul Republic of Korea; 4 Biomedical Engineering Research Center Asan Institute for Life Sciences Asan Medical Center Seoul Republic of Korea; 5 Department of Digital Health Samsung Advanced Institute for Health Sciences & Technology SungKyunKwan University Seoul Republic of Korea; 6 Big Data Research Center Samsung Medical Center Seoul Republic of Korea; 7 Cancer Big Data Center National Cancer Center Gyeonggi-do Republic of Korea

**Keywords:** data anonymization, privacy act, data sharing, data protection, data linking, health care data demand

## Abstract

**Background:**

There has been significant effort in attempting to use health care data. However, laws that protect patients’ privacy have restricted data use because health care data contain sensitive information. Thus, discussions on privacy laws now focus on the active use of health care data beyond protection. However, current literature does not clarify the obstacles that make data usage and deidentification processes difficult or elaborate on users’ needs for data linking from practical perspectives.

**Objective:**

The objective of this study is to investigate (1) the current status of data use in each medical area, (2) institutional efforts and difficulties in deidentification processes, and (3) users’ data linking needs.

**Methods:**

We conducted a cross-sectional online survey. To recruit people who have used health care data, we publicized the promotion campaign and sent official documents to an academic society encouraging participation in the online survey.

**Results:**

In total, 128 participants responded to the online survey; 10 participants were excluded for either inconsistent responses or lack of demand for health care data. Finally, 118 participants’ responses were analyzed. The majority of participants worked in general hospitals or universities (62/118, 52.5% and 51/118, 43.2%, respectively, multiple-choice answers). More than half of participants responded that they have a need for clinical data (82/118, 69.5%) and public data (76/118, 64.4%). Furthermore, 85.6% (101/118) of respondents conducted deidentification measures when using data, and they considered rigid social culture as an obstacle for deidentification (28/101, 27.7%). In addition, they required data linking (98/118, 83.1%), and they noted deregulation and data standardization to allow access to health care data linking (33/98, 33.7% and 38/98, 38.8%, respectively). There were no significant differences in the proportion of responded data needs and linking in groups that used health care data for either public purposes or commercial purposes.

**Conclusions:**

This study provides a cross-sectional view from a practical, user-oriented perspective on the kinds of data users want to utilize, efforts and difficulties in deidentification processes, and the needs for data linking. Most users want to use clinical and public data, and most participants conduct deidentification processes and express a desire to conduct data linking. Our study confirmed that they noted regulation as a primary obstacle whether their purpose is commercial or public. A legal system based on both data utilization and data protection needs is required.

## Introduction

There has been considerable effort to use health care data [1,2], and many countries have implemented regulations to protect the privacy of patients and research subjects [3-5]. Owing to the sensitivity of health care data, privacy protection laws have limited its use [6]. Regulations that focus only on protecting privacy are emerging as a major challenge in using health care data [7-9].

Health care institutions and governments both generate a large amount of heterogeneous data [10]. To use these decentralized data, there have been dramatic increases in linking data from diverse sources [11]. By using big data analytic approaches, which leverage data drawn from multiple sources [12], data-driven research has the potential for widespread positive impact and global implications [13-16]. Efforts have been made to use health care data for the following purposes: ensuring a high level of evidence by using a large number of samples [17], identifying risk factors [18], and improving diagnosis and treatment standards [19].

However, in Korea, this use conflicts with the current regulations because data linking requires the data be identified and shared [20,21]. The privacy law of Korea is known as the strongest principle in Asia [22-24]. Although most discussions about privacy laws have centered on data protection, discussions about the privacy law are now about the need to facilitate the development of industries that utilize data beyond protection [8]. However, there has been no mention of what makes data usage and deidentification processes difficult or users’ needs for data linking from a practical perspective.

The objective of this study is to investigate (1) the status of big data utilization in different medical areas (general hospitals, universities, industry, and academic society); (2) institutional obstacles and efforts in deidentification processes, which is an alternative approach for using health care data; and (3) users’ data linking needs.

## Methods

### Study Design and Data Collection

This study is designed to investigate the demand for health care data, identify the difficulties in using health care data, and develop improvements for using health care data from the practical users’ perspective. For this, we conducted a cross-sectional online survey. To recruit participants who use health care data, we (1) publicized the survey promotion campaign through social media (Facebook) and (2) sent official documents to academic societies encouraging participation in the online survey. Through the provided documents, anyone who used health care data was able to participate in the questionnaire (online open survey; see details in Multimedia Appendix 1).

The online questionnaire was developed and distributed using Office forms (Naver, Korea). This questionnaire could be accessed from mobile phones and personal computers. To ensure important questions were answered, seven mandatory items were designated among the 17 questions. This function was used to prevent participants from submitting responses without checking the answers on mandatory items before submission. However, the questionnaire did not verify data consistency. For example, respondents who replied that they did not have a demand for health care data could also select “clinical data” as a response to the question asking about required data. To ensure the validity of the questionnaire, the items on the questionnaire were developed through 15 revisions in consultation with eight experts over a period of approximately one month. The final questionnaire consisted of 16 items within five parts. Each screen contained one to eight questions; there were a total of eight screens in the survey (on mobile and PC screens).

Ethical clearance was obtained from the Public Institutional Review Board designated by The Korean Ministry of Health and Welfare (number: 2018-2199-001) before data collection.

### Participant Recruitment

We selected five academic societies (Korean Society of Medical Informatics, Korean Society for Preventive Medicine, Korean Cancer Association, Korea Society of Artificial Intelligence in Medicine, and Korean Society of Epidemiology) that exhibit a high demand for health care data or were recommended by experts. Then, we encouraged participation in the survey by sending an official letter requesting cooperation for online surveys to the secretariat of each academic society.

A web link to access the survey was provided to interested respondents. Respondents were required to provide consent through this link. To receive consent from respondents, the first screen of the online questionnaire included the background, purpose, and duration of the research, as well as a description of the disadvantages or limitations. After respondents approved this introduction, the link led to the anonymous online questionnaire. As an incentive for participation, they were offered coffee gift vouchers by submitting their cell phone numbers. To transfer the coffee vouchers and exclude duplicate responses, informed consent to collect cell phone numbers was received separately. The cross-sectional online survey was conducted between October 5 and 19, 2018.

By the end of the survey period, 128 participants responded to the online survey. Responses that were contradictory (n=2) or did not exhibit a demand for health care data (n=8) were excluded; therefore, a total of 118 participants were included in the analysis. The overall eligible population of subjects was unknown because the online survey was sent to the five academic societies and was advertised through a social media promotion.

Among the responses (N=118), quality improvement of welfare services and research promotion were considered to be public purposes (81/115, 70.4%) and industrial development and profit generation were classified as commercial purposes (34/115, 29.6%); this classification excludes other minor purposes (n=3).

### Questionnaire Items

The survey items were categorized into five parts. The first part included items that investigated the work experience and basic information of participants. The second part inquired about the type of data participants wanted. The third part related to obstacles and improvement suggestions for data use. The fourth part investigated the identification process, and the last part investigated data linking (details in Multimedia Appendix 2).

### Statistical Analysis

Analyses were conducted using R (version 3.5.1) and Microsoft Excel (version 2016). Descriptive statistics for proportions of respondents, work profiles (eg, age, work experience, expertise area, working institution), and responses regarding data demand, data linking, and deidentification were explored.

For categorical variables, such as data needs, obstacles, and improvement suggestions, chi-square tests were performed to show these reponses were different between participants using data for public purposes and those using data for commercial purposes. We conducted chi-square tests with one section as the response to specific questions, such as obstacles to using health care data. Chi-square tests could not be performed for responses to questions that allowed participants to choose more than one answer (multiple response questions), such as data needs, because the responses were not independent. For questions that could have multiple responses, post hoc chi-square tests were performed (Multimedia Appendices 3 and 4). Post hoc pairwise chi-square tests involved testing each value of the nominal variable versus the sum of all others. After applying the same principle of chi-square to get the *P* value for each comparison, we then used Bonferroni correction to counteract the problem of type I error that occurs when multiple comparisons are made.

## Results

### Overall Population

The majority of online survey participants worked in a general hospital (62/118, 52.5%; multiple response question) or university (51/118, 43.2%; multiple response). Most participants were in the field of research (84/118, 71.2%), in their thirties (56/118, 47.5%), and had work experience between 1 and 5 years (56/118, 47.5%; Table 1).

**Table 1:** Profile of online survey respondents (N=118).

Characteristics		Respondents, n (%)
**Age (years)**		
	20-29	21 (17.8)
	30-39	56 (47.5)
	40-49	34 (28.8)
	50-59	5 (4.2)
	Other	2 (1.7)
**Institution (multiple response question)**		
	General hospital	62 (52.5)
	University	51 (43.2)
	Industry	15 (12.7)
	Academic society	6 (5.1)
	Other	3 (2.6)
**Expertise**		
	Research	84 (71.2)
	Data analysis	18 (15.3)
	Planning	11 (9.3)
	Device development	5 (4.2)
**Expertise experience**		
	≥10 years	9 (7.6)
	5 years to <10 years	32 (27.1)
	1 year to <5 years	56 (47.5)
	<1 year	21 (17.8)

### Data Demand, Obstacles, and Improvement Suggestions

More than half of participants replied that they had a need for clinical data (82/118, 69.5%) and public data (76/118, 64.4%; Table 2). Only the general hospital group selected clinical data in a high proportion (56/62, 90.3%).

Participants reported that the most significant obstacles in trying to use health care data were conflicts with the law (53/118, 44.9%) and data standardization (50/118, 42.4%). However, the obstacles most frequently selected by each group were different. Overall, the four groups of respondents by institution (general hospital, university, industry, and academic society) reported data standardization problems and legal conflicts as the main challenges in using data.

Similarly, most participants indicated that legislation improvement was required to overcome these data utilization limitations (54/118, 45.8%), followed by the need for technical measures for data standards (47/118, 39.8%). Overall, participants suggested that law revision was the first priority of improvement (Table 2).

There was no statistically significant difference in the percentage of obstacles in groups that used health care data for either commercial or public purposes (*P*=.38). However, both groups indicated that data standardization and current laws function as constraints of health care data use (Table 3).

**Table 2:** Data needs, obstacles, and developmental proposals for data utilization.

Characteristics		Respondents, n (%)					Total (N=118), n (%)
		General hospital (n=62)	University (n=51)	Industry (n=15)	Academic society (n=6)	Other (n=3)	
**Data needs (multiple response question)**							
	Clinical data (collected during care process in hospital)	56 (90.3)	29 (56.9)	10 (66.7)	5 (83.3)	0 (0.0)	82 (69.5)
	Public data (managed by nation)	40 (64.5)	32 (62.7)	10 (66.7)	5 (83.3)	3 (100.0)	76 (64.4)
	Research data (clinical research or trial data)	38 (61.3)	33 (64.7)	3 (2.0)	5 (83.3)	0 (0.0)	61 (51.7)
	Life log data (patient generated health data)	17 (27.4)	16 (31.4)	8 (53.3)	4 (66.7)	1 (33.3)	36 (30.5)
	Genetic data	13 (21.0)	18 (35.3)	1 (6.7)	2 (33.3)	1 (33.3)	28 (23.7)
**Obstacle**							
	Conflict of laws	30 (48.4)	19 (37.3)	8 (53.3)	2 (33.3)	0 (0.0)	53 (44.9)
	Data standardization	24 (38.7)	23 (45.1)	5 (33.3)	3 (50.5)	3 (100.0)	50 (42.4)
	Strict social recognition	5 (8.1)	4 (7.8)	2 (13.3)	1 (16.7)	0 (0.0)	9 (7.6)
	Other	1 (1.6)	1 (4.0)	0 (0.0)	0 (0.0)	0 (0.0)	2 (1.7)
	None	2 (3.2)	3 (5.9)	0 (0.0)	0 (0.0)	0 (0.0)	4 (3.4)
**Improvement suggestion (multiple response question)**							
	Law revision	29 (46.8)	22 (43.1)	7 (46.7)	4 (66.7)	2 (66.7)	54 (45.8)
	Technical measures	22 (35.5)	23 (43.1)	6 (40.0)	3 (50.0)	1 (33.3)	47 (39.8)
	Utilization support	12 (19.4)	7 (13.7)	0 (0.0)	0 (0.0)	0 (0.0)	16 (13.6)
	Public consensus	6 (9.7)	11 (21.6)	2 (13.3)	1 (16.7)	1 (33.3)	22 (18.6)

**Table 3:** Comparison of data demand, obstacles, and improvement suggestions between health care data use for commercial and public purposes (N=115). Sample size excludes the n=3 for other minor purposes.

Measures		Respondents, n (%)		Chi-square (df)	*P* value
		Public purpose (n=81)	Commercial purpose (n=34)		
**Data needs (multiple response question)**				Not applicable	Not applicable
	Clinical data	53 (65.4)	28 (82.4)		
	Public data	53 (65.4)	23 (67.6)		
	Research data	44 (54.3)	16 (47.1)		
	Life log data	18 (22.2)	18 (52.9)		
	Genetic data	17 (21.0)	9 (26.5)		
**Obstacles**				2.9 (4)	.38
	Conflict with laws	38 (46.9)	13 (38.2)		
	Data standardization	35 (43.2)	15 (44.1)		
	Strict social recognition	6 (7.4)	3 (8.8)		
	None	1 (1.2)	2 (5.9)		
	Other	1 (1.2)	1 (2.9)		
**Improvement (multiple response question)**				Not applicable	Not applicable
	Law revision	38 (46.9)	15 (44.1)		
	Technical method	33 (40.7)	14 (41.2)		
	Data utilization support	15 (18.5)	1 (2.9)		
	Public consensus	14 (17.3)	6 (17.6)		

### Deidentification

When using health care data, 101 participants responded that they conduct deidentification measures (101/118, 85.6%). The majority of participants reported that multiple deidentification methods are used (64/101, 63.4%). The most common method was pseudonymization (72/101, 71.3%), followed by masking (57/101, 56.4%). Most respondents who conducted deidentification considered privacy issues induced by rigid social culture as the biggest problem for deidentification (28/101, 27.7%), followed by the absence of clear criteria for deidentification measures (24/101, 23.8%; Table 4).

**Table 4:** Responses about the current state of data deidentification (N=118).

Measures		Respondents, n (%)
**Deidentify when using health care data (n=118)**		
	Yes	101 (85.6)
	No	17 (14.4)
**Number of applied deidentification methods (n=101)**		
	1 method	37 (31.4)
	2 methods	33 (28.0)
	3 methods	18 (15.3)
	4 methods	4 (3.4)
	5 methods	9 (7.6)
**Applied methods (n=101; multiple response question)**		
	Pseudonymization	72 (71.3)
	Masking	57 (56.4)
	Data reduction	37 (36.6)
	Data suppression	30 (29.7)
	Aggregation	22 (21.8)
**Difficulties when deidentifying data (n=101)**		
	Strict social culture	28 (27.7)
	Absence of clear deidentification guideline	24 (23.8)
	Usefulness of deidentified data	15 (14.9)
	Lack of understanding of deidentification policy and technology	14 (13.9)
	Lack of relevant institution support	11 (10.9)
	Lack of deidentification measure for unstructured data	9 (8.9)

### Data Linkage

The majority of participants answered that they require data linking (98/118, 83.1%). The difference in the proportion of respondents who wanted to use data linkage for public or commercial purposes was not statistically significant (*P*=.64). The 98 respondents who said that data linking was necessary indicated that the purpose of linking data was to obtain longitudinal data (62/98, 63.3%). In addition, deregulation and data standardization comprised a large proportion of data linking improvement suggestions (33/98, 33.7% and 38/98, 38.8%, respectively). In the two items that investigated the reason for data linkage and suggestions to facilitate data linking, the proportion of responses in both the public purpose and commercial purpose groups did not significantly differ (*P*=.16 and *P*=.47, respectively).

The groups that used data for public purposes responded that health care data are to be used to develop health care policy (41.8%, 28/81). On the other hand, the group that used data for commercial purposes primarily responded that data was to be used for the development of diagnostic technology (n=12; Table 5).

**Table 5:** Demand for health care data linking.

Measures		Participants, n (%)				Chi-square (df)	*P* value^a^
		Public purpose (n=81)	Commercial purpose (n=34)	Other (n=3)	Total N=118)		
**Data linking**						0.2 (1)	.64
	Required	67 (82.7)	30 (88.2)	1 (33.3)	98 (83.1)		
	Not required	14 (17.3)	4 (11.8)	2 (66.7)	20 (16.9)		
**Reason for data linking (n=98)**						3.6 (2)	.16
	Obtain longitudinal data	39 (58.2)	23 (76.7)	0 (0.0)	62 (63.3)		
	Obtain larger number of subjects	15 (22.4)	5 (16.7)	0 (0.0)	20 (20.4)		
	Develop policy predicated on data	13 (19.4)	2 (6.7)	1 (100.0)	16 (16.3)		
**Suggestions for facilitating health care data linking (n=98)**						2.5 (3)	.47
	Deregulation	22 (32.8)	11 (36.7)	0 (0.0)	33 (33.7)		
	Data standardization	28 (41.8)	10 (33.3)	0 (0.0)	38 (38.8)		
	Effective guidelines including procedure, responsibility, and technology	11 (16.4)	8 (26.7)	1 (100.0)	20 (20.4)		
	Improvement of social recognition	6 (9.0)	1 (3.3)	0 (0.0)	7 (7.1)		
**Usage details** **(n=98)**						18.8 (6)	.003
	Development of health care policy	28 (41.8)	2 (6.7)	1 (100.0)	31 (31.6)		
	Development of diagnostic technology	15 (22.4)	12 (40.0)	0 (0.0)	27 (27.6)		
	Development of treatment modality	12 (17.9)	4 (13.3)	0 (0.0)	16 (16.3)		
	General research	8 (11.9)	4 (13.3)	0 (0.0)	12 (12.2)		
	Development of medical device	2 (3.0)	6 (20.0)	0 (0.0)	8 (8.2)		
	Development of new drug	1 (1.5)	1 (3.3)	0 (0.0)	2 (2.0)		
	Other	1 (1.5)	1 (3.3)	0 (0.0)	2 (2.0)		
	Subtotal	67 (100.0)	30 (100.0)	1 (100.0)	98 (100.0)		

^a^Public versus commercial.

## Discussion

### Principal Findings

The primary finding of this study was the clarification of each health care area’s need for data. Most wanted to use clinical data and public data, except for university respondents. Considering the amount of stored data depending on the health care field [25], it is understandable that clinical data are in high demand.

Secondly, most participants who use health care data conduct deidentification measures before data use. The majority of deidentification measures are implemented using more than one method. This survey was not able to distinguish between cases in which deidentification was not conducted when required and cases in which it was not conducted because the data was not identifiable (whether due to the exclusion of personal information or the lack of legal deidentification requirements).

Although it is not clear whether these respondents voluntarily implemented deidentification measures or were obligated to do so, it appears they consider health care data to be sensitive information. Their use of multiple deidentification measures may be considered proof of action to mitigate concerns about privacy infringement. However, they pointed out that rigid social culture acts as a primary obstacle in data deidentification. Therefore, if we prove that privacy is guaranteed, we can achieve social consensus and relieve sociocultural rigidity.

Lastly, the proportion of respondents who need to link data was significantly larger than the proportion of respondents who do not; these respondents indicated that deregulation and standardization are necessary to facilitate data linkage. This suggests that many of these respondents face difficulties due to intensive regulation. Data users may experience legal conflicts when they want to link data from external data sources. When linking with external data, an identifier is required, which is often personal information. If consent has been obtained for other research purposes previously, this identifier can be used; however, in big data analysis, there are limitations on obtained consent [21]. Furthermore, for personal information to be provided to third parties, they must obtain the consent of the subjects (article 17, Personal Information Protection Act [PIPA]). Practically, it has been burdensome for controllers to recontact individual subjects and obtain consent; thus, they may be obstructed by law [26]. Data sharing and linkage are limited by the PIPA [27].

In recent years, some countries have attempted to revise their information protection legislation to prepare for the development of a new information industry [28,29]. The United States has enacted the Final Rule, a revision of the Common Rule, to reduce the regulatory burden and create a new concept of broad consent to enhance both the use and protection of data [4,30]. The European Union, by enacting the General Data Protection Regulation, has strengthened data protection principles while including principles such as the right to data portability [31]. In Japan, the concept of anonymizing processed information is defined by law, and the use of personal information is being promoted [32].

Yet, privacy remains an issue in countries that are trying to implement centralized electronic health records (EHRs), such as Canada. Centralized EHRs could have interoperability in terms of data structure because the same data schema enables data linking and communication. This would reduce the obstacle for health care data use. However, in terms of comprehensive use and communication of data, the privacy issue must be handled for secondary use. Therefore, as long as privacy remains an issue, there will also be a need for data linking. For example, a study on a Canada-wide EHR system stated that privacy systems should address the issues of deidentifying health care data and privacy concerns [33,34]; skeptics have warned against adopting a Canada-wide EHR system until then [35]. Furthermore, even with centralized EHRs, the privacy issue will remain in situations of linking with privately collected data, such as mobile data and data collected by wearable devices. In a survey conducted in the United States to identify digital health adoption and sentiments of consumers, results showed people are rarely willing to provide personal health data to pharmaceutical companies, research institutes, or information technology companies [36].

Considering this global trend, the regulation of personal information in Korea does not reflect these changes [37]. There have been many studies on methods by which regulation can be improved to reflect changes in secondary data usage; however, to provide a basis for these legislative improvements, there was a need for evidence to show that actual users experienced these difficulties and needs for data linking.

The results of this study confirm that the use of health care data conflicts with the law, which leads to the implication that legislation should be revised to facilitate data utilization. However, it should not simply be deregulated, but balanced between protection and utilization, as is the case of major countries. To improve this legal system, a survey of opinions on the use of health care data also should be conducted on the data supplier and beneficiary side (the general population). In the United States, these surveys about digital health consumer’s sentiments have been conducted, and most respondents remain wary about sharing their health data with technology companies [36]. Likewise, surveys on how the opinions of hospital’s data managers differ from those of the users in our study should be conducted to achieve a better social consensus and reconcile the two areas of data utilization and protection.

### Limitations

The respondents in this study were primarily involved in general hospitals and universities, whereas the respondents in academia and industry were few. Respondents affiliated with universities are considered to hold concurrent positions in general hospitals. In addition, we did not obtain significant information on the characteristics of the entire population in the survey because survey promotion was conducted through social network services and the transmission of official documents. However, considering the number of medical institutions in Korea (tertiary hospitals or secondary hospitals) and the number of universities, it is natural that many respondents belong to medical institutions and universities. Although this may not directly represent the opinion of the entire population in need of health care data, in the absence of previous studies that directly investigate the opinions of data users, this study has the advantage of illuminating the present status of Korean data users’ perspectives in a cross-sectional way.

In addition, to represent the overall opinion of the population, it is necessary to select the population for each institution and extract a sample using a stratified sampling method. We examined the current circumstances of health care data use from data users’ perspectives, but data managers and beneficiaries should also be surveyed for policy development to ensure that all parties are considered in bridging the gap between data privacy and utilization.

### Conclusion

This study provides a cross-sectional view from a practical user-oriented perspective on the types of data users find valuable, the efforts and obstacles that characterize deidentification processes, and users’ needs for data linking. Most respondents seek to use clinical and public data. Moreover, most implement deidentification measures. We confirmed that they want to link data but are limited by regulations regardless of whether their purpose is commercial or public. A legal system that is founded on both the utilization and protection of data is necessary.

## Appendix

Multimedia Appendix 1 Notice for survey.

Multimedia Appendix 2 Online questionnaire on health care data utilization.

Multimedia Appendix 3 Post hoc pairwise chi-square test for comparison of data needs, obstacles, and improvement.

Multimedia Appendix 4 Post hoc chi-square test results: demand for health care data linking.

## Abbreviations

EHR: electronic health record
PIPA: Personal Information Protection Act
