# Supplementary figures and images for: Why Do Data Users Say Health Care Data Are Difficult to Use? A Cross-Sectional Survey Study
Source: J Med Internet Res. 2019 Aug 6;21(8):e14126. doi: 10.2196/14126 (PMC6701164; doi:10.2196/14126)

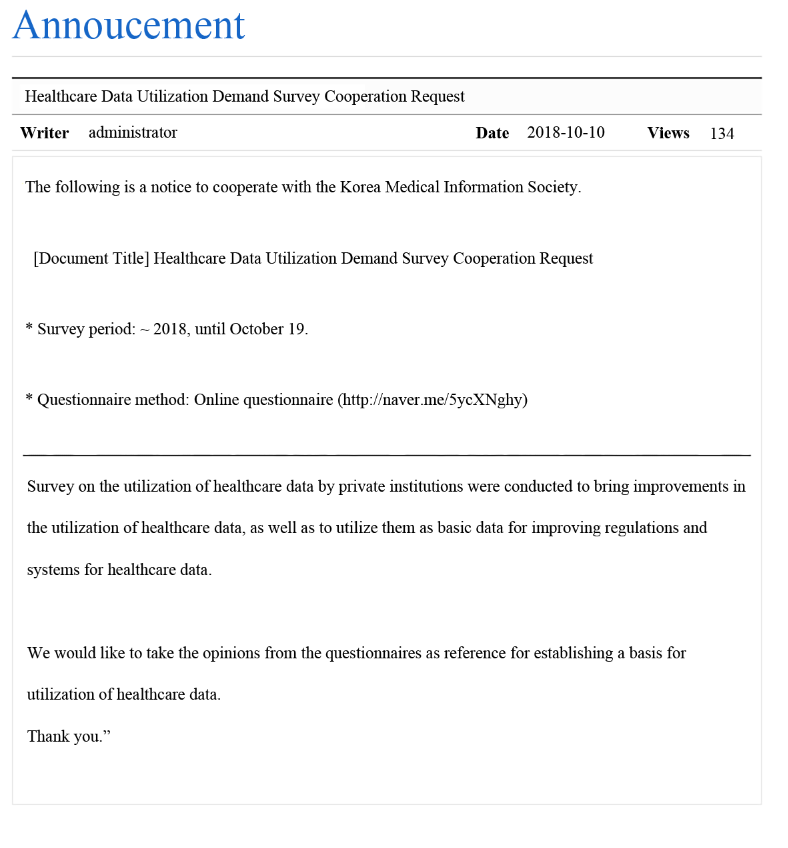

Supplement: Multimedia Appendix 1 [file jmir_v21i8e14126_app1.png]
