# Supplementary material for: Why Do Data Users Say Health Care Data Are Difficult to Use? A Cross-Sectional Survey Study
Source: J Med Internet Res. 2019 Aug 6;21(8):e14126. doi: 10.2196/14126 (PMC6701164; doi:10.2196/14126)
Supplement: Multimedia Appendix 2 [file jmir_v21i8e14126_app2.docx]

The online questionnaire of survey about the healthcare data utilization

| 1. Which institutions do you belong to? (multiple)   □ Hospital □ University □ Industry □ Academic society □ Other ( )   1. What type of healthcare data do you want to use?   □ Clinical data: data generated from patient care processes such as diagnosis history, treatment data, order history, admission note, nursing records, and laboratory results  □ Genetic data: such as microarray data, NGS data, and human genome polymorphism  □ Public data: such as NHI claims data  □ Clinical trial data: data generated from clinical trials and studies  □ Life log data: data captured automatically by wearable technology and mobile devices  □ None   1. (Question 2, continued) Which institutions have the data you want to use?  \| Healthcare data \| Sources \| \| --- \| --- \| \| - Clinical data \| □ In your institution (hospital or industry)  □ Outside your institution (hospital or industry)  □ Other ( ) \| \| - Genetic data \| □ In your institution (hospital or industry)  □ Outside your institution (hospital or industry)  □ Other ( ) \| \| - Public data \| □ Korea National Health Insurance Service  □ Korea National Cancer Center (cancer cohort registry)  □ Korea Health Insurance Review and Assessment Service  □ Korea Center for Disease Control and Prevention  □ Other ( ) \| \| - Research data \| □ In your institution (hospital or industry)  □ Outside your institution (hospital or industry)  □ Korea Center for Disease Control and Prevention  □ Other ( ) \| \| - Life log data \| □ In your institution (hospital or industry)  □ Outside your institution (hospital or industry)  □ Korea National Health Insurance Service  □ Korea Center for Disease Control and Prevention  □ Other ( ) \|  1. (Question 2, continued) For which purpose will the data be used?   □ Development of diagnostic technology  □ Development of treatment modality  □ Development of new drug  □ Development of medical devices  □ Development of healthcare policy  □ General research  □ Other ( )   1. (Question 2. continue) Which one is an expected effect of using data?   □ Improvement of public and health service quality  □ Development of the healthcare industry  □ Activation of health and medical research  □ Creation profits by developing new product and service  □ Other ( )   1. (Question 2. continue) Which obstacles do you face while using data?   □ Data standardization  □ Conflict of laws principles  □ Strict social recognition  □ None  □ Other ( )  7. Which problem must be improved in order to facilitate data utilization?  □ Law system reform: personal information protection laws improvement  □ Technical support: infrastructure improvement for health data standardization  □ Data utilization support: consultation such as legal advice, de-identification, and analysis  □ Public consensus: need to address the public’s concerns, such as providing visible national benefits and enhancing security systems  □ Other ( )  8. Does the data you want to use require healthcare data linkage?  □ Required  □ Not required (go to Question 11)  9. For which reasons do you need data linkage?  □ To get the larger number of subjects  □ To get longitudinal data (medical history) about patients who visit several hospitals  □ To make policy predicated on data  □ Other ( )  10. Which is the most important thing to activate healthcare data linkage? (Multiple)  □ Data standardization  □ Deregulation (Apply negative regulation)  □ Improvement of social recognition  □Actually an effective guideline that describe legal procedure, technical reference and responsibility  □ Other ( )  11. Do you de-identify when you use healthcare data?  □ Yes  □ No  13. Choose the methods you use when you de-identify data (Multiple)  □ Pseudonymization  □ Aggregation  □ Data reduction  □ Data suppression  □ Data masking  14. Which obstacles do you face when you de-identify data?  □ Understanding de-identification policy and technique  □ Absence of de-identification guidelines for each data type  □ Absence of de-identification institutional support  □ Strict social culture induced privacy concerns  □ Lack of de-identification technique  □ Value of de-identified data  [Profile survey]  1. Choose your age range  □ 20-29 □ 30-39 □ 40-49 □ 50-59 □ other  2. Which area is your expertise?  □ Research  □ Data analysis  □ Planning  □ Development of Healthcare device  □ other ( )  3. How many years of work experience do you have in health care?  □ None  □ Less than 1 year  □ 1 year or more and less than 5 years  □ 5 years or more and less 10 years  □ Above 10 years |
| --- | --- | --- | --- | --- | --- | --- | --- | --- | --- | --- | --- | --- |
