# Supplementary material for: Why Do Data Users Say Health Care Data Are Difficult to Use? A Cross-Sectional Survey Study
Source: J Med Internet Res. 2019 Aug 6;21(8):e14126. doi: 10.2196/14126 (PMC6701164; doi:10.2196/14126)
Supplement: Multimedia Appendix 3 [file jmir_v21i8e14126_app3.docx]

Post-hoc pairwise chi-square test for comparison of data needs, obstacles, and improvement

|  | Respondents, % (total n=115) | | Chi-square test  Adjusted *P*-value |
| --- | --- | --- | --- |
| Measures | Public purpose  (n = 81) | Commercial purpose  (n=34) |  |
| **Data needs, n (%), multiple choices** |  |  |  |
| Clinical data | 53 (65.4) | 28 (82.4) | 1.0 |
| Public data | 53 (65.4) | 23 (67.6) | 1.0 |
| Research data | 44 (54.3) | 16 (47.1) | 1.0 |
| Life log data | 18 (22.2) | 18 (52.9) | 1.0 |
| Genetic data | 17 (21.0) | 9 (26.5) | .21 |
| **Obstacles, n (%)** |  |  |  |
| Conflict with laws | 38 (46.9) | 13 (38.2) | 1.0 |
| Data standardization | 35 (43.2) | 15 (44.1) | 1.0 |
| Strict social recognition | 6 (7.4) | 3 (8.8) | 1.0 |
| None | 1 (1.2) | 2 (5.9) | 1.0 |
| Other | 1 (1.2) | 1 (2.9) | 1.0 |
| **Improvement, n (%), multiple choices** |  |  |  |
| Law revision | 38 (46.9) | 15 (44.1) | 1.0 |
| Technical method | 33 (40.7) | 14 (41.2) | 1.0 |
| Data utilization support | 15 (18.5) | 1 (2.9) | .39 |
| Public consensus | 14 (17.3) | 6 (17.6) | 1.0 |
