# Supplementary material for: Why Do Data Users Say Health Care Data Are Difficult to Use? A Cross-Sectional Survey Study
Source: J Med Internet Res. 2019 Aug 6;21(8):e14126. doi: 10.2196/14126 (PMC6701164; doi:10.2196/14126)
Supplement: Multimedia Appendix 4 [file jmir_v21i8e14126_app4.docx]

the results of post-hoc for chi-square test: Demand for healthcare data linking

| **Measures** | Participants (n, %) | | | | Chi-square test  Adjusted *P-*value  (Public vs. Commercial) |
| --- | --- | --- | --- | --- | --- |
|  | Public purpose  (n=81) | Commercial purpose  (n=34) | Other  (n=3) | Total  (n=118) |  |
| **Data linking** |  |  |  |  | .64 |
| Required | 67 (82.7) | 30 (88.2) | 1 (33.3) | 98 (83.1) |  |
| Not required | 14 (17.3) | 4 (11.8) | 2 (66.7) | 20 (16.9) |  |
| **Reason for data linking (n=98)** |  |  |  |  |  |
| Obtain longitudinal data | 39 ( 58.2) | 23 ( 76.7) | 0 ( 0.0) | 62 (63.3) | .38 |
| Obtain larger number of subjects | 15 ( 22.4) | 5 ( 16.7) | 0 ( 0.0) | 20 (20.4) | 1.0 |
| Develop policy predicated on data | 13 ( 19.4) | 2 ( 6.7) | 1 (100.0) | 16 (16.3) | .58 |
| **Suggestions for facilitating healthcare data linking (n=98)** |  |  |  |  |  |
| Deregulation | 22 ( 32.8) | 11 ( 36.7) | 0 ( 0.0) | 33 (33.7) | 1.0 |
| Data standardization | 28 ( 41.8) | 10 ( 33.3) | 0 ( 0.0) | 38 (38.8) | 1.0 |
| Effective guidelines including procedure, responsibility, and technology | 11 ( 16.4) | 8 ( 26.7) | 1 (100.0) | 20 (20.4) | 1.0 |
| Improvement of social recognition | 6 ( 9.0) | 1 ( 3.3) | 0 ( 0.0) | 7 (7.1) | 1.0 |
| **Usage details (n=98)** |  |  |  |  |  |
| Development of healthcare policy | 28 ( 41.8) | 2 ( 6.7) | 1 (100.0) | 31(31.6) | <.01 |
| Development of diagnostic technology | 15 ( 22.4) | 12 ( 40.0) | 0 ( 0.0) | 27 (27.6) | 1.0 |
| Development of treatment modality | 12 ( 17.9) | 4 ( 13.3) | 0 ( 0.0) | 16 (16.3) | 1.0 |
| General research | 8 ( 11.9) | 4 ( 13.3) | 0 ( 0.0) | 12 (12.2) | 1.0 |
| Development of medical device | 2 ( 3.0) | 6 ( 20.0) | 0 ( 0.0) | 8 (8.2) | 1.0 |
| Development of new drug | 1 ( 1.5) | 1 ( 3.3) | 0 ( 0.0) | 2 (2.0) | 1.0 |
| Other | 1 ( 1.5) | 1 ( 3.3) | 0 ( 0.0) | 2 (2.0) | 1.0 |
| Sub total | 67 (100.0) | 30 (100.0) | 1 (100.0) | 98(100.0) |  |
